# The *PPO* family in *Nicotiana tabacum* is an important regulator to participate in pollination

**DOI:** 10.1186/s12870-024-04769-3

**Published:** 2024-02-09

**Authors:** Xuemei Wei, Keliang Tao, Zhengmei Liu, Boyuan Qin, Jie Su, Yanbi Luo, Chunwen Zhao, Jugou Liao, Junpeng Zhang

**Affiliations:** 1 School of Engineering, Dali University, Dali, 671000 Yunnan Province China; 2 School of Life Science, Biocontrol Engineering Research Center of Plant Diseases & Pests, Biocontrol Engineering Research Center of Crop Diseases & Pests, Yunnan University, Kunming, 650091 Yunnan Province China

**Keywords:** *Nicotiana tabacum*, Polyphenol oxidase, Pollen tube growth, Pollination

## Abstract

Polyphenol oxidases (PPOs) are type-3 copper enzymes and are involved in many biological processes. However, the potential functions of PPOs in pollination are not fully understood. In this work, we have screened 13 *PPO* members in *Nicotiana. tabacum* (named *NtPPO1-13*, *NtPPOs*) to explore their characteristics and functions in pollination. The results show that NtPPOs are closely related to PPOs in *Solanaceae* and share conserved domains except NtPPO4. Generally, *NtPPO*s are diversely expressed in different tissues and are distributed in pistil and male gametes. Specifically, *NtPPO9* and *NtPPO10* are highly expressed in the pistil and mature anther. In addition, the expression levels and enzyme activities of *NtPPOs* are increased after *N. tabacum* self-pollination. Knockdown of *NtPPOs* would affect pollen growth after pollination, and the purines and flavonoid compounds are accumulated in self-pollinated pistil. Altogether, our findings demonstrate that *NtPPO*s potentially play a role in the pollen tube growth after pollination through purines and flavonoid compounds, and will provide new insights into the role of PPOs in plant reproduction.

**Supplementary Information:**

The online version contains supplementary material available at 10.1186/s12870-024-04769-3.

## Introduction

Polyphenol oxidases (PPOs) are common nuclear-coded copper-containing enzymes found in all insects, fungi, vertebrates, and plants [1, 2]. PPOs belong to the oxidoreductase family and are mainly located in the thylakoid membrane of chloroplast [3]. Generally, PPOs can be categorized into catechol oxidase (EC 1.10.3.1), tyrosinase (E.C.1.14.18.1) and laccase (EC 1.10.3.2) according to their substrate specificity and structure [3]. Catechol oxidases catalyze two distinct reactions involving molecular oxygen: the o-hydroxylation of monophenols to o-diphenols (cresolase activity) and the oxidation of o-diphenols to o-quinones (catecholase activity). Tyrosinases always catalyze the hydroxylation of monophenols, and laccases oxidize o-diphenols and p-diphenols to form their corresponding quinones [2].

In plant tissue, PPOs may exist in a latent form containing transit peptides, and they are activated by proteolytic cleavage until they are incorporated into the plastid [2, 4]. Transit peptides generally contain three evident regions: an uncharged amino-terminal domain, a central domain lacking acidic residues, and a carboxy-terminal domain with the potential to form an amphiphilic P-strand [5]. Transit peptides with conserved domains have been found in most of the identified PPOs [5].

PPOs are a class of oxidoreductases among the six major defensive enzymes, and play important roles in defense mechanisms, and also have function in metabolic pathways and biosynthesis [6, 7]. Antisense down-regulation of *PPOs* expression can result in hyper-susceptibility to pathogens, suggesting that PPO-mediated phenolic oxidation plays a critical role in plant defense [8]. Over-expressing the *PPOs* in tomato plants increases their resistance to *P. syringae* [9]. Wound- and herbivore-induced expression of *PPOs* in hybrid poplar supports the defensive role of this protein against insect pests [10, 11]. In the phenylpropanoid pathway, PPOs are assumed to be responsible for hydroxylation of p-coumaric acid to caffeic acid [12]. In the creosote bush (*Larrea tridentata*), PPOs have a central role in the biosynthesis of the creosote bush 8–8’ linked lignans, which have potent antiviral, anticancer, and antioxidant properties [13]. Aureusidin synthase (the plant polyphenol oxidase family) specifically catalyzes the oxidative formation of aurones, and plays a key role in the yellow coloration of snapdragon flowers [14–16]. The biosynthesis of flavones in MicroTom tomato is related to a tomato polyphenol oxidase (SlPPOF), which possesses a flavone synthase-like activity [17]. In addition, PPOs are also involved in betalain biosynthesis [18], and contribute to several biological processes, including plant development [19, 20], cell differentiation and death [21, 22], mehler reaction, electron cycling and oxygen regulation [23].

Previous studies have shown that the reproductive signaling pathways might co-evolve with that of defense responses, the interaction between the pollen and the style shared similarities to that of bacterial defense responses [24], such as the majority of known defensing-like (DEFLs) proteins have been confirmed to be predominately expressed in reproductive organs, and play numerous roles in reproduction [22]. As the member of plant defense systems, one *PPO* member (*tobP1*) is reported to be specially expressed in the flower of tobacco [25]. Furthermore, *PPOs* exert an important role in pollen development through modulating flavonoids homeostasis in tobacco [26]. These studies suggest that *PPOs* play a significant role in plant reproduction. However, the potential functions of *PPOs* in the pollination are, as of yet, unclear.

In this work, we identify the characteristics of *PPO* family in *Nicotiana* (an essential model system for studying the reproduction and development), detect their tissue expression profile and distribution, and explore their potential function and mechanism in pollination. Our research will contribute to deepen the scientific knowledge on *PPOs* in *Nicotiana*, and provide new insights into the role of *PPOs* in plant reproduction.

## Materials and methods

### Plant materials

In this study, RNAi lines against all *NtPPOs* and wild-type (WT, the allotetraploid cultivar *N. tabacum L.* K326 (2n = 24II = 48TTSS)) plants from Biocontrol Engineering Research Center of Plant Diseases & Pests, Yunnan University were seeded in humus and cultured in a climatic room (28 °C, 16 h light, 8 h dark) for about 7–8 weeks. All plants were transplanted into soil in the greenhouse with average temperature of 25 °C and natural sunlight, watered twice a week, and fertilized with water-soluble fertilizer (N:P:K = 20:20:20 + 0.5% trace elements) (Demei, Sichuan, China) once a month.

### Multiple sequence alignment and phylogenetic analysis

NtPPO protein sequences were retrieved from the genomic data of *N. tabacum* using HMM searching program. PPO proteins from other species were retrieved from NCBI. Multiple sequence alignments of the all protein sequences were carried out using ClustalW in MEGA 7.0 [27]. A phylogenetic tree was constructed using MEGA 7.0 with the maximum-likelihood (ML) method and bootstrap values was set to 1000 [27]. The phylogenetic tree was estimated using iTOL [28].

### Analysis of cis-acting elements in *NtPPOs* promoters

The promoter sequences (upstream regions of ATG, 1.5-kb) [41, 42] of *NtPPOs* were obtained from NCBI, and the cis-acting regulatory elements were identified by PlantCARE [29]. Visualization was performed using GraphPad Prism 7 (Inc., San Diego, CA, USA).

### RNA extraction and reverse transcription-quantitative real-time PCR

Total RNA was extracted from the root, stem, leaf, pistil, mature anther (stage8) [30] using MiniBEST Plant RNA Extraction Kit (Takara, Dalian, China). The cDNA was reverse transcribed by PrimeScript™ RT reagent Kit with gDNA Eraser (Perfect Real Time) (Takara). All of the operational procedures were performed following the manufacturer’s protocols. For further RT-qPCR analysis, 1 µL of cDNA was diluted with 9 µL of nuclease-free water. Gene-specific primer pairs were designed by Primer Premier 5.0 and tested by NCBI Primer BLAST for specificity, and the products were 80 to 200 bp in length (Table S1). All RT-qPCR were conducted using Super Real PreMix Plus (SYBR Green) (TIANGEN, Beijing, China) with a total volume of 10 µL reaction system on the ABI 7500 Real-Time PCR System. Ubiquitin-conjugating enzyme E2 (Ntubc2) was used as an internal reference, as it has been proven to express itself at relatively stable levels in different tissues [31]. Relative expression levels were calculated using the 2^−ΔΔCT^ method [32]. The data was processed with GraphPad 7.0, with means ± standard deviation (SD). Three technical replicates were used for each of the three biological replicates.

### Antibodies preparation

A monoclonal antibody library against the pistil and anther of *Nicotiana* was constructed according to the previously described [33]. A total of 300 *N.tabacum* flowers from five plants were manually emasculated before anthesis, and 150 were pollinated with *N.tabacum* pollen and *Nicotiana. stocktonii*, respectively. The pistils (without ovary) were collected for protein extraction at 10 h after pollination (HAP). Total proteins were extracted as previous report [34]. Antibody microarray screening and processing of mAb array data were performed according to the previously described [33]. Then, the expression patterns of proteins with the most significant differences in the antibody slides were analyzed further by western blot (WB). Immunoprecipitation (IP), and liquid chromatography mass spectrometry/mass-spectrum (LCMS/MS) assays were performed as previously described [35]. The LCMS/MS data was searched against the NCBI *N.tabacum* and Hyoscyamus niger cybrid databases, containing 1, 520, 274 entries. IP-LC-MSMS assays showed that one of the differentially expressed proteins was PPO. Therefore, the monoclonal antibody against PPO (anti-mouse IgG) was successfully screened and identified from the monoclonal antibody library of *Nicotiana*. Theoretically, the antibody can recognize all of NtPPOs. The secondary antibody IgG H&L (ab150113, Alexa Fluor®488) was obtained from Abmart (Shanghai, China).

### Sample preparation and immunofluorescence microscopy

Approximately 40 *N.tabacum* flowers were emasculated before anthesis and artificially pollinated with *N.tabacum* pollen. The flowers were then incubated at 28 °C, and ten pollinated pistils were collected at 2.5, 5, 10, and 15 HAP. Mature anthers, fresh pollen grains, *N.tabacum* un-pollinated pistils were collected. These samples were immediately fixed with 4% paraformaldehyde in 20 mM PBS (pH 7.4). After fixation for 24 h, samples were dehydrated in an ethanol series, rinsed several times with cacodylate buffer, embedded in LR White resin, and sectioned as previously described [36].

Before IF staining, a series of treatments were performed. First, the sections were dried at 65 °C for 30 min, de-waxed with xylene, rehydrated with different concentrations of ethanol and distilled water, then washed three times with 1× PBS buffer. After treating with 200µL of endogenous peroxidase potent blocking solution (Beyotime P0100B) for 10 min at 25 °C, the sections were boiled in 1×antigen repair solution (Beyotime P0083) for antigen repair and blocked with immunostaining blocking solution (Beyotime P0260) for 10 min. Next, the slides were incubated with the primary antibody (1: 500) at 4 °C for 12 h. After the antibody was washed off with 1× PBS buffer, the slides were incubated with a second antibody (1: 400, Abmart ab150113, Alexa Fluor ® 488) for 3 h at 25 °C and washed five times with 1× PBS buffer. Finally, the slides were observed on an Olympus BX51 (Olympus, Shinjuku, Tokyo, Japan) and photographed with an Olympus DP73 (Olympus, Shinjuku, Tokyo, Japan). For the control, the primary antibody was replaced with 1× PBS buffer.

To detect the expression and localization of *NtPPOs* in pollen tubes, the fresh and mature pollen grains were cultivated on the adherent slide with pollen germination medium (10% sucrose, 0.005% boric acid, and 0.08% agarose) for 3 h at 28 °C. The medium was naturally air-dried for 24 h, and the slide was subjected to IF staining.

### NtPPOs activity assays

Pistils of *N.tabacum* were pollinated artificially with *N.tabacum* pollen, and collected at 2.5, 5, 10 and 15 HAP. Then, the total proteins in these samples were isolated respectively. The total protein isolation and NtPPOs activity assays were performed following the PPO enzyme activity assay kit protocol (Solarbio, Beijing, China) using L-3, 4-dihydroxy-Phe (L-DOPA) as substrate.

### Semi in vitro test and aniline blue dying

WT and transgenic lines were self-pollinated and incubated at 28 °C for 5, 8 and 12 h. Some pollinated pistils were collected at 5 and 8 HAP, and softened in 1 M NaOH for 3 h at 60 °C, then dyed with 0.1 M aniline blue in 0.1 M K_2_HPO_4_ for more than 2 h. Pistils were squashed and observed under a fluorescence microscope (Olympus BX51) [37]. Remaining pollinated pistils (12 HAP) were cut from the middle of the style with a sharp blade and immediately immersed into a pollen germination medium (10% sucrose, 0.005% boric acid, and 0.08% agarose) on glass slides. The samples continue to be cultivated for 12 h at 28 °C, and pollen tubes growing out of the cutting end of pistil were observed under an Olympus BX51 (Olympus, Japan).

### Untargeted metabolomics analysis

Self-pollinated *Nicotiana* pistils with six biological replicates for metabolomics analysis of WT and *NtPPO-RNAi* lines were collected at 24 HAP and immediately frozen in liquid nitrogen and stored at − 80 °C, un-pollinated pistils served as control. Then gas chromatography-mass spectrometry, data processing and analysis, and screening for metabolic differences were performed as previously described [38].

### Statistical analysis

Homoscedasticity of variances were tested in SPSS, and then the data were analyzed using GraphPad Prism 7 (Inc., San Diego, CA, USA). Student’s t-test or ANOVA was performed to detect statistical differences.

## Results

### Phylogenetic and conserved domain analysis of the PPO family in *N. tabacum*

**Fig. 1:**
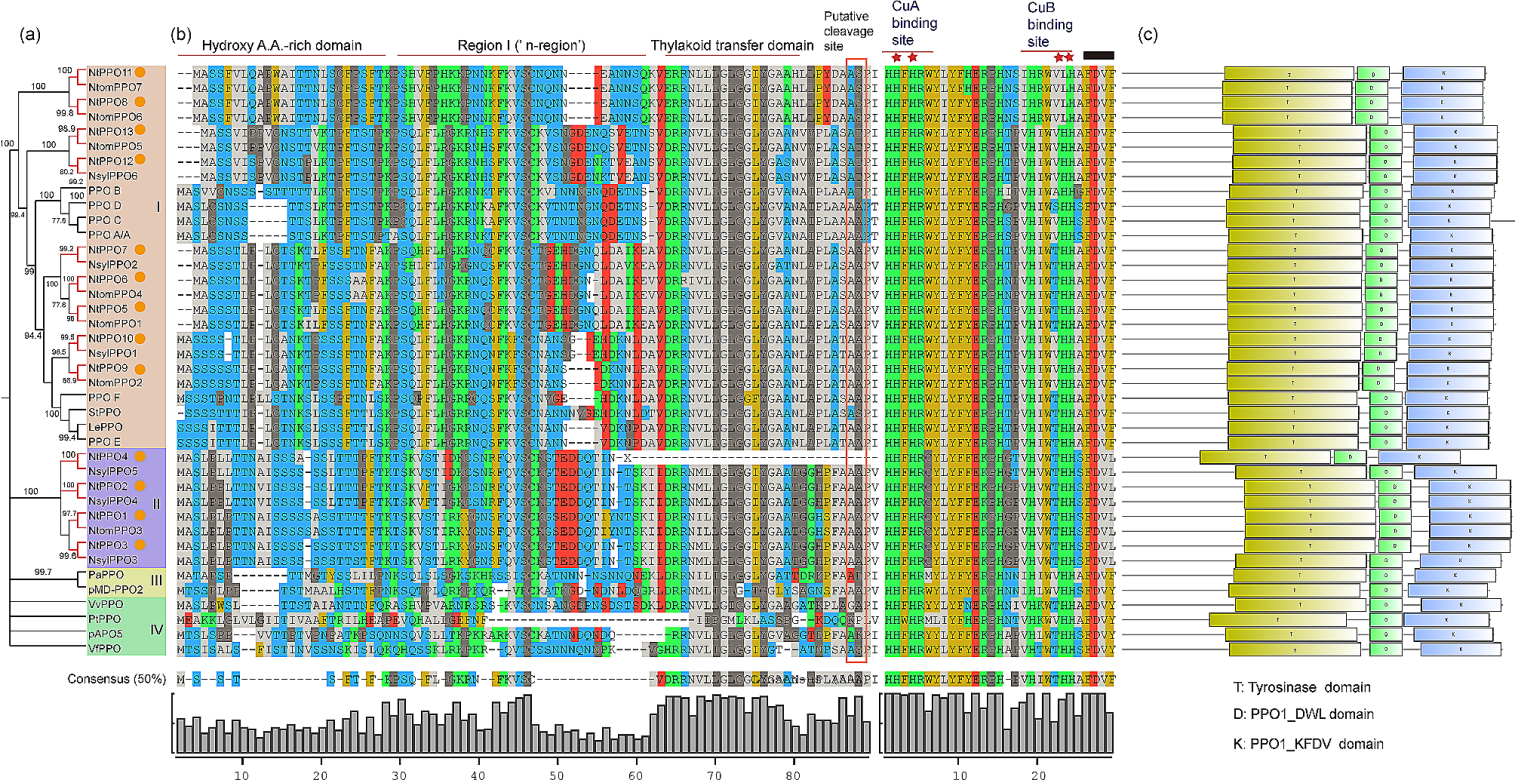
Phylogenetic tree and conserved domains of PPOs in *N. tabacum* and other plant species. (**a**) Phylogenetic tree of NtPPOs and other PPOs in different plant species. PPOs are divided into four groups (I, II, III, IV). NtPPO members are marked with an orange circle. Red branches indicate the pairs of paralogous PPOs. PPO sequences obtained from other plant species genomes are as follows: *Fugi apple* pMD-PPO2, *Solanum lycopersicum* PPO A/A’, PPOB, PPOC, PPOD, PPOE, PPOF, apple pAPO5, *Lycopersicon esculentum* LePPO, *Solanum tuberosum* StPPO, *Vicia faba* VfPPO, *Vitis vinifera* VvPPO, *Prunus armeniaca* PaPPO, *Populus trichocarpa* PtPPO. Ntom, *Nicotiana tomentosoformis L*.; Nsyl, *Nicotiana sylvestris L*. (**b**) Putative transit peptide and function domain analysis by multiple sequence alignment. Gaps were introduced to achieve maximum homology among the sequences. The hydroxy A.A.-rich, Region I (‘n-region’) and thylakoid transfer domains are underlined with red lines, respectively. Putative cleavage sites are marked by a red box. His-rich copper binding domains CuA, CuB are underlined with red lines. His residues predicted to be copper-binding sites are marked by red star. KFDV domain is marked with black squares. (**c**) Conserved domain analysis. T, tyrosinase domain; D, PPO1_DWL domain; K, PPO1_KFDV domain

Our previous study screened 13 *NtPPO* members in the *N. tabacum* genome designated *NtPPO1-13* [26]. To understand their evolutionary relationships, an un-rooted phylogenetic tree was constructed using the NtPPO sequences, PPOs from their ancestor genomes and published PPOs from other plants (Table S2). Bootstrap values and phylogenetic relationships divided these PPOs into four groups (I, II, III, IV), and the NtPPO members were distributed in group I (nine NtPPOs) and II (four NtPPOs) (Fig. 1a). NtPPOs and PPOs from their ancestor genomes formed paralogous pairs, indicating that NtPPO5, NtPPO6, NtPPO8, NtPPO9, NtPPO11 and NtPPO13 were originated from the female parent-*Nicotiana sylvestris*, and NtPPO1, NtPPO2, NtPPO3, NtPPO4, NtPPO7, NtPPO10 and NtPPO12 were traced back to the male parent-*Nicotiana tomentosoformis* (Fig. 1a). PPOs in *Solanum lycopersicum* gemome (PPO A/A’, PPOB, PPOC, PPOD, PPOE, PPOF), *Lycopersicon esculentum* genome (LePPO), and *Solanum tuberosum* genome (StPPO) were assigned to the same group as NtPPOs (group I) (Fig. 1a), indicating the close relationship between them. Other PPOs (pMD-PPO2, pAPO5, VfPPO, VvPPO, PaPPO, PtPPO) and NtPPOs were in different groups, suggesting a distant genetic relationship (Fig. 1a).

PPOs are nuclear-encoded copper-containing enzymes that move from the cytoplasm to thylakoid lumen of the chloroplast, and finally localize in the plastids [4, 39]. As is typical for thylakoid lumen proteins, PPOs may exist in the latent form containing ubiquitous transit peptides at their N-terminal. These transit peptides have conserved domain structures, and are activated by proteolytic cleavage [40]. We further analyzed the conserved domain of NtPPOs by multiple sequence alignment, and found that most of NtPPO members showed common structural characteristics except NtPPO4 (Fig. 1b). The N-terminal of NtPPOs was a hydroxy A.A.-rich domain, and contained many hydroxylated Ser and Thr residues, but very few acidic amino acids (Fig. 1b). The highly conserved thylakoid transfer domains (the typical feature of thylakoid lumen protein) were distributed in NtPPOs, and Region I (‘n-region’) was located between the Hydroxy A.A.-rich and thylakoid transfer domains (Fig. 1b). The predicting putative cleavage sites were presented between Ala and Asp (Fig. 1b). Particularly, we have found that NtPPO4 had no thylakoid transfer domain and putative cleavage site (Fig. 1b). There were two His-rich copper binding domains (CuA, CuB) distributed in the protein sequences responsible for the catalytic function of NtPPO proteins (Fig. 1b). Furthermore, all of NtPPOs shared conserved domains, including tyrosinase domain, PPO1_DWL domain and PPO1_KFDV domain (Fig. 1c).

### Diversity of cis-acting elements in the promoter regions of *NtPPO*s

**Fig. 2:**
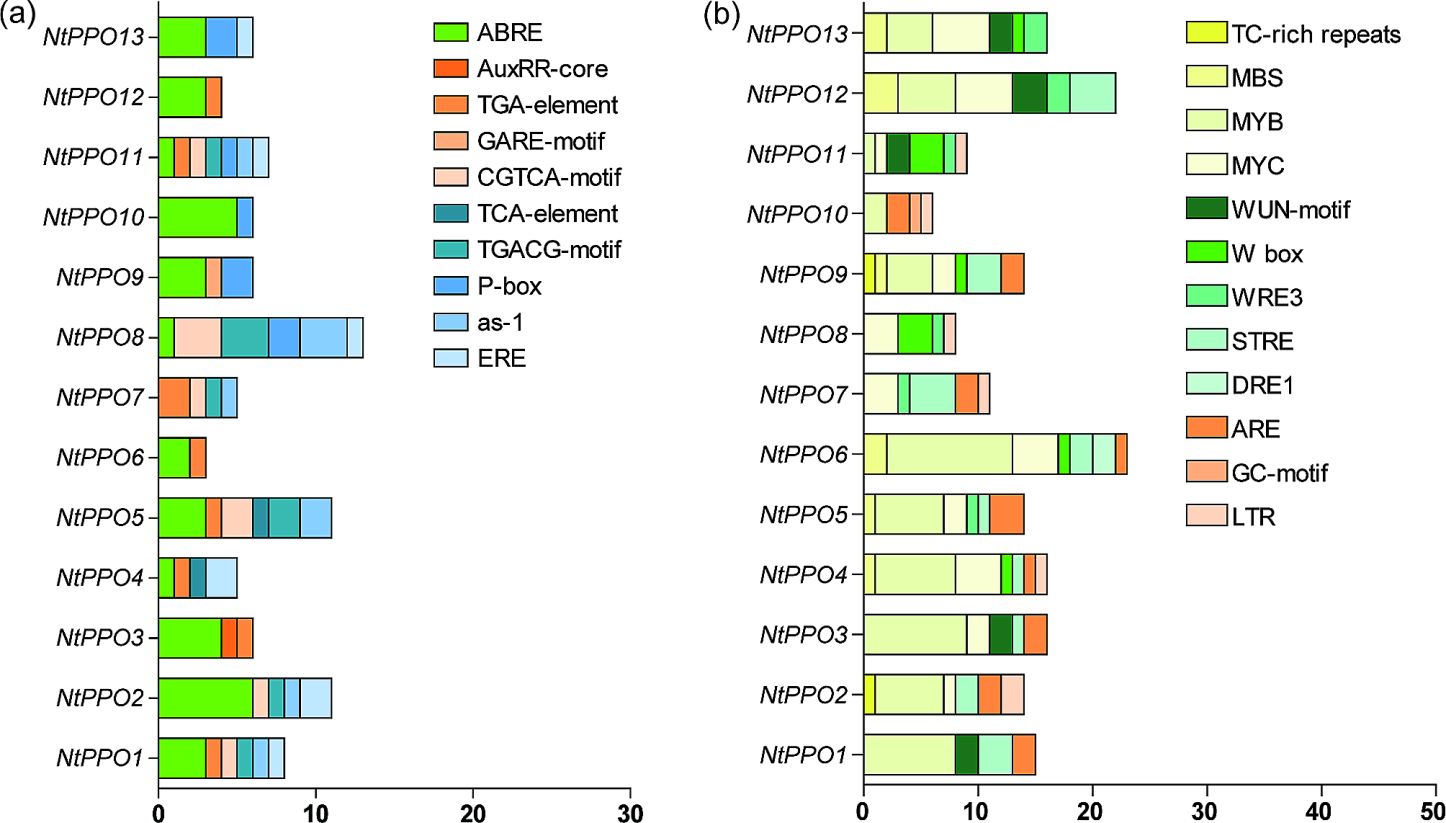
Function prediction of the cis-elements detected in the *NtPPO* promoters. (**a**) Predicted plant hormone-responsive elements in *NtPPOs* promoter regions. (**b**) Predicted stress-inducible and defense-related elements in *NtPPOs* promoter regions

To study the potential regulatory mechanisms involved in *NtPPO* transcriptions, the 1.5-kb upstream promoter regions from the translation start sites of all of *NtPPO* members were analysed with PlantCARE [29]. Ten plant hormone-responsive elements and twelve abiotic stress-response-related elements were predicted, and their distribution in promoters of *NtPPOs* was analysed and displayed (Fig. 2, Table S3). The 10 plant hormone-responsive elements were distributed in the most of *NtPPO* members, including abscisic acid responsiveness element (ABRE), auxin-responsive elements (TGA-element), MeJA-responsive element (CGTCA-motif, TGACG-motif), ethylene-responsive element (ERE), auxin and salicylic acid responsiveness element (as-1), and gibberellin-responsive elements (P-box) (Fig. 2a). In addition, salicylic acid responsiveness element (TCA-element), auxin-responsive elements (AuxRR-core) and other gibberellin-responsive elements (GARE-motif,) were also found in the promoters of *NtPPOs* (Fig. 2a). Abiotic stress-response-related elements—such as MBS, MYB, MYC, WUN-motif, WRE3, W-box, STRE, ARE, and LTR were abundant in *NtPPO* promoters (Fig. 2b). Furthermore, defence and stress responsiveness elements (TC-rich repeats) were also found in the promoters of some *NtPPO* members (Fig. 2b). The above results display that the expression of *NtPPOs* may be associated with hormone signals and environmental stress.

### Expression profiles of the *NtPPOs* are likely to be tissue-specific

**Fig. 3:**
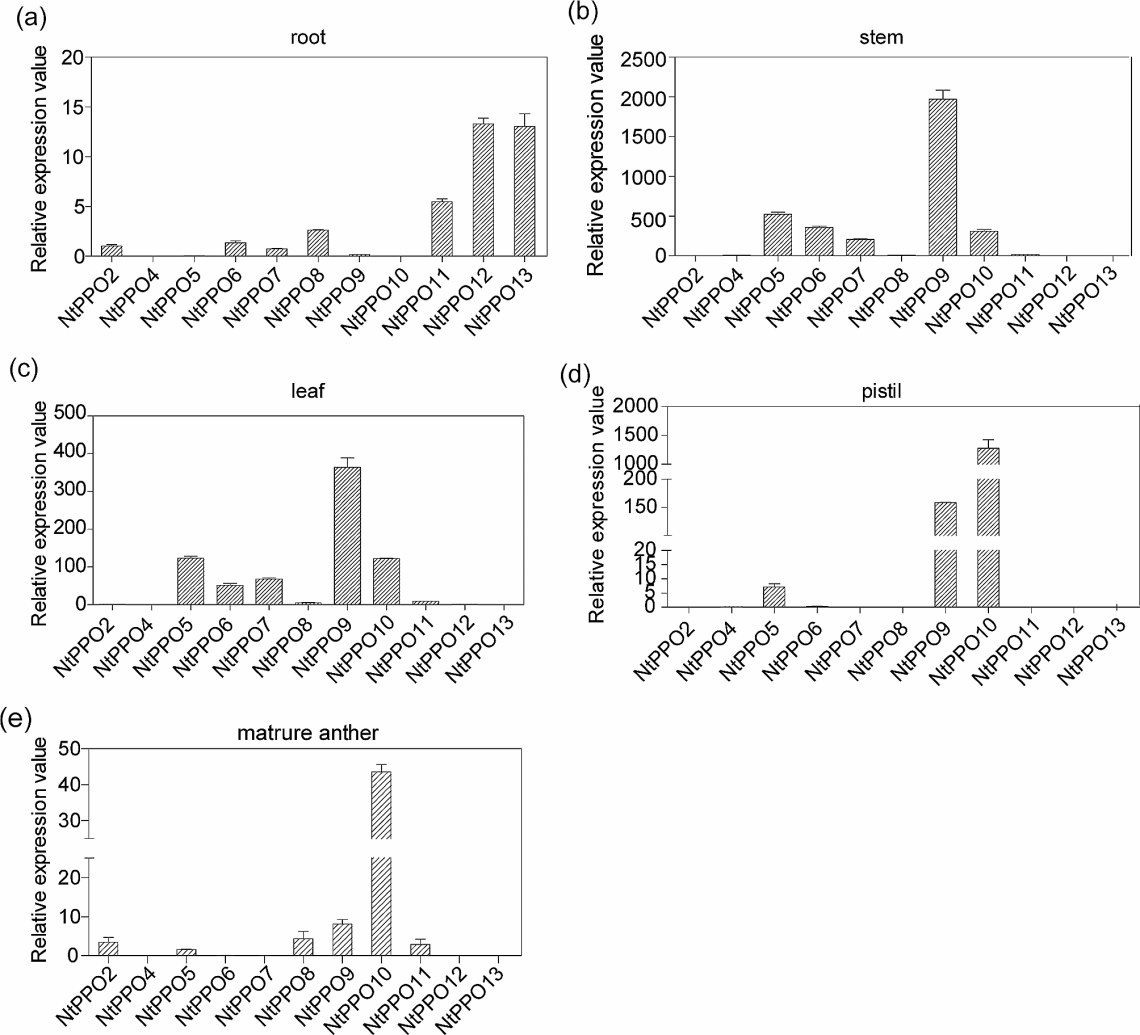
The expression profiles of *NtPPO* members in different tissues. RT-qPCR detcetion of *NtPPOs* expression in the root, stem, leaf, pistil and mature anther of *N.tababum*

In order to investigate the tissue specificity of *NtPPOs* in *Nicotiana* tissues, we detected the expression profiles of *NtPPO* members in the root, stem, leaf, pistil, and mature anther of *N.tababum* by reverse transcription-quantitative real-time (RT-qPCR). The results showed that, except *NtPPO1* and *NtPPO3*, 11 out of 13 *NtPPO*s can be detected in *N.tababum* tissues (Fig. 3). The expression levels of *NtPPO2* and *NtPPO4* were relatively low in various tissues, while *NtPPO12* and *NtPPO13* had the highest relative expression in the root in comparison with the other *NtPPO* members (Fig. 3a). In the stem and leaf, the expression levels of *NtPPO5, NtPPO6, NtPPO7, NtPPO9* and *NtPPO10* were high (Fig. 3b, c). Particularly, *NtPPO9* showed the highest expression levels in the stem and leaf. Moreover, the expression levels of *NtPPO9* and *NtPPO10* were also high in the pistil and mature anthers, especially *NtPPO10* (Fig. 3d and e). It is noted that NtPPO9 (A0A1S3YVF2) and NtPPO10 (A0A1S4B7N5) were the only two PPO members identified in the *N.tabacum* self-pollinated pistil by IP-LC-MS/MS analysis (Table S4). These results indicate that *NtPPO9* and *NtPPO10* are the most likely the candidate *PPO* members functioning in the pistil and pollen, and may play a role in pollen tube growth and pollination.

### *NtPPOs* are abundantly expressed in the reproductive tissues

**Fig. 4:**
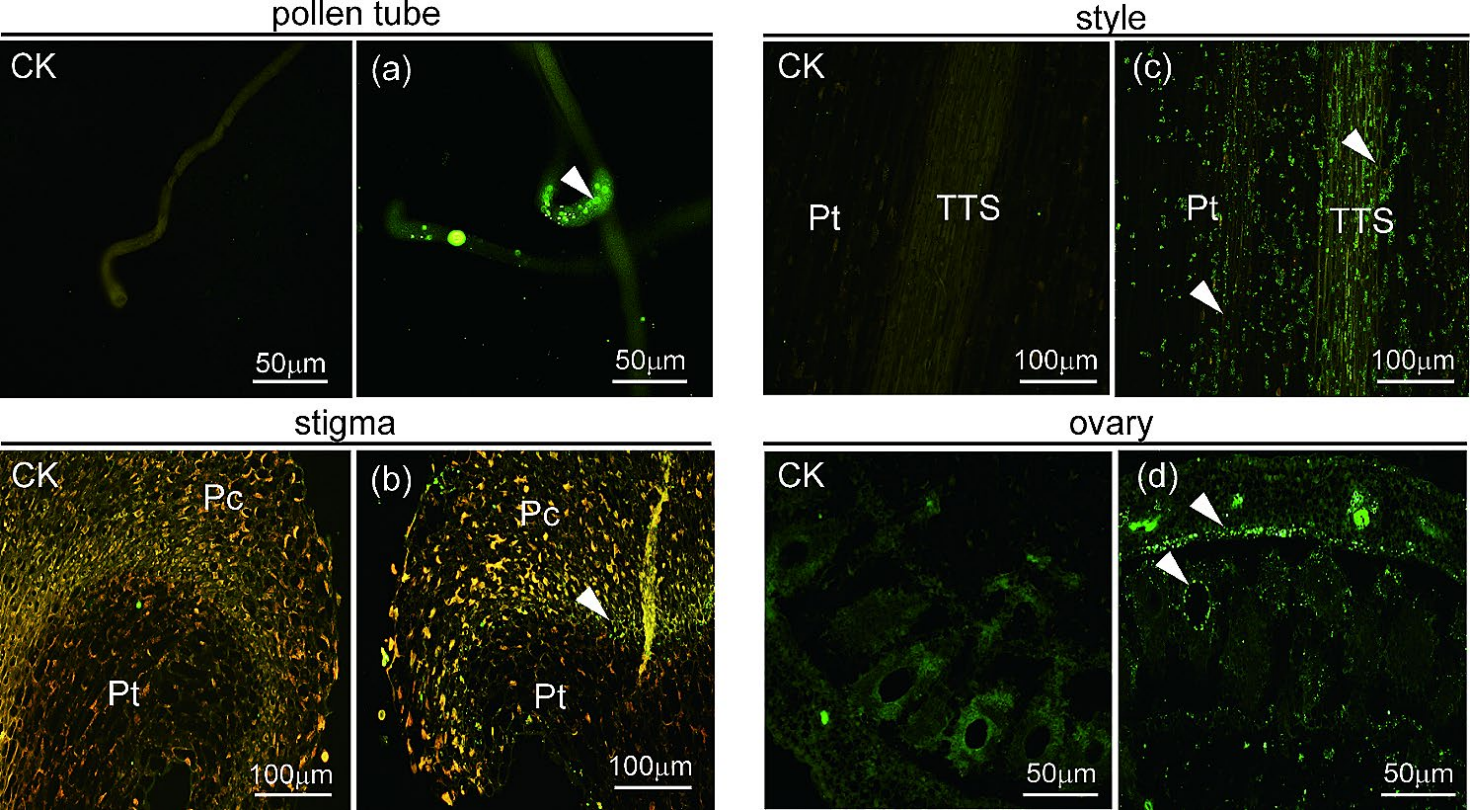
Expression and distribution analysis of *NtPPOs* in the reproductive tissues by IF. For negative control, the primary antibody was replaced with 1×PBS buffer. (**a**) In vitro growing pollen tubes. The arrow indicates NtPPOs in the pollen tube tip. (**b**) Stigma micrograph. The arrow shows the NtPPOs in the interface of the papillae cell layer and transmitting tissue. (**c**) Style micrograph. The arrow shows the NtPPOs in the transmitting tissue and parenchymatous tissue. (**d**) Ovary micrograph. The arrow shows NtPPOs in the ovary wall and ovule. Ct, connective tissue; Aw, anther wall; Pc, papillae cell; Pt, parenchymatous tissue; TTS, transmitting tissue

The expression and distribution of *PPOs* in different tissues provided possible clues to the function of them in these tissues. By using immunofluorescence microscopy (IF), we further explored the expression and distribution of *NtPPOs* in the reproductive tissues. Strong green fluorescence signals were observed at the tips of the growing pollen tubes (in the apical zone and subapical (or clear region) and shank regions (or shear region) (Fig. 4a). In the stigma, weak green fluorescence signals were detected in the papillae cell layers (Fig. 4b). *NtPPOs* were expressed in the trans-mitting tissue (TTS) and parenchymatous cell layers of the stigma and style (Fig. 4c). In the ovary, green fluorescence signals were observed in the ovary wall and ovule, and were mainly accumulated surrounding the periphery of the embryo sac (Fig. 4d). In the pistil and pollen tubes, the rich expression and wide distribution of *NtPPOs* demonstrated their potential function in pollination.

### The expression of *NtPPOs* are activated in self-compatible stigma/style

**Fig. 5:**
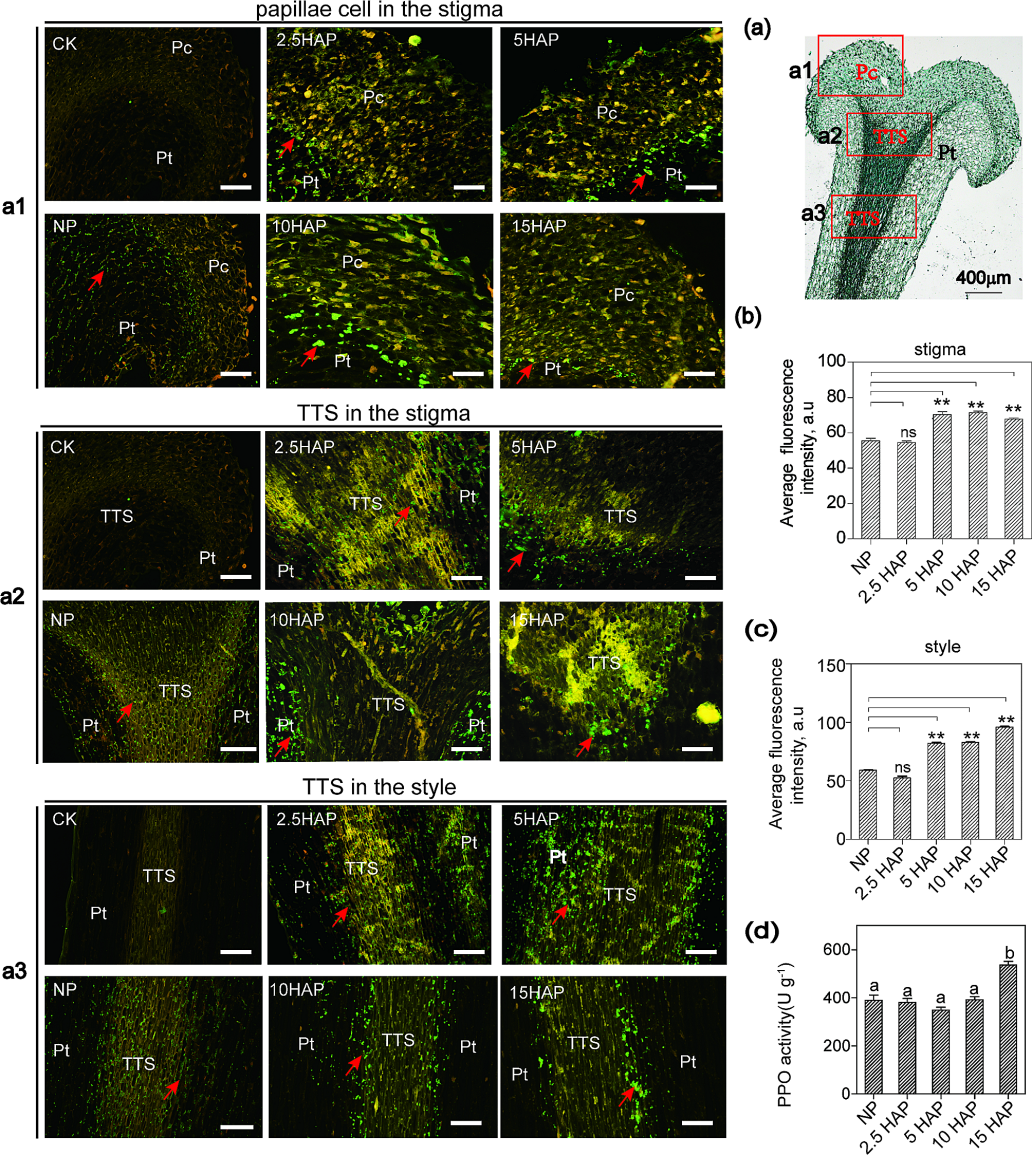
Expression and distribution analysis of *NtPPOs* in the *N.tabtacum* self-pollinated stigma/style. For negative control, the primary antibody was replaced with 1×PBS buffer. (**a**) The morphology of stigma and style in *N.tabtacum* and IF analysis of NtPPO contents and distribution in the un-pollinated stigma/style, and in the *N.tabtacum* self-pollinated stigma/style at 2.5, 5, 10, and 15 HAP. NP, un-pollinated; HAP, hours after pollination; Pc, papillae cell; TTS, transmitting tissue; Pt, parenchymatous tissue. The red box indicates the observation sites. Red arrow indicates NtPPO fluorescent signals. Scale bar: 50 μm. (**b-c**) Quantification of green fluorescence signals intensity. The error bar represents standard deviation from three repeats, ** *P* < 0.01, Student’s t-test. (**d**) NtPPOs activity in the *N.tabtacum* NP stigma/style and self-pollinated stigma/style.Three biological replicates were performed. Error bars with different letters are significantly different from each other (*P* < 0.05). Data were analyzed by ANOVA

In order to further explore if *NtPPOs* play a role in pollen tube growth after pollination, their expression in the *N.tabacum* self-pollinated (self-compatible) stigma/style was detected via IF and enzyme activity assay at 2.5, 5, 10, and 15 h after pollination (HAP). The results showed that the difference of NtPPO signals (green fluorescence) intensity between un-pollinated stigma/style and self-pollinated stigma/style at 2.5 HAP was not significant, and NtPPO signals in the un-pollinated stigma/style were weaker than that in the self-pollinated stigma/style at 5, 10, and 15 HAP (Fig. 5a1-a3, b and c). In the stigma, NtPPO signals were rarely observed in the papillae cell layer, and were mainly distributed in the parenchymatous tissue (Fig. 5a1). NtPPO signals were distributed in the TTS of stigma at 2.5, 5 and 15 HAP (Fig. 5a2). Interestingly, after 10 h of pollination, NtPPO signals were rarely detected in the TTS, while distributed at the boundaries of the TTS (Fig. 5a2). In the style, NtPPOs were mainly distributed at the boundaries of the TTS, and rarely detected in the middle of the TTS (Fig. 5a3). The enzyme activity of NtPPOs in the *N.tabacum* self-pollinated pistil was the highest at 15 HAP (Fig. 5d). These results indicated that the expression of *NtPPO*s was activated after pollination, and the NtPPOs were accumulated more and the enzyme activity was also increased after pollination, and further corroborated the potential role of *NtPPOs* in pollen tube growth after pollination.

### Knockdown of *NtPPOs* affects pollen growth after pollination and fruit weights

**Fig. 6:**
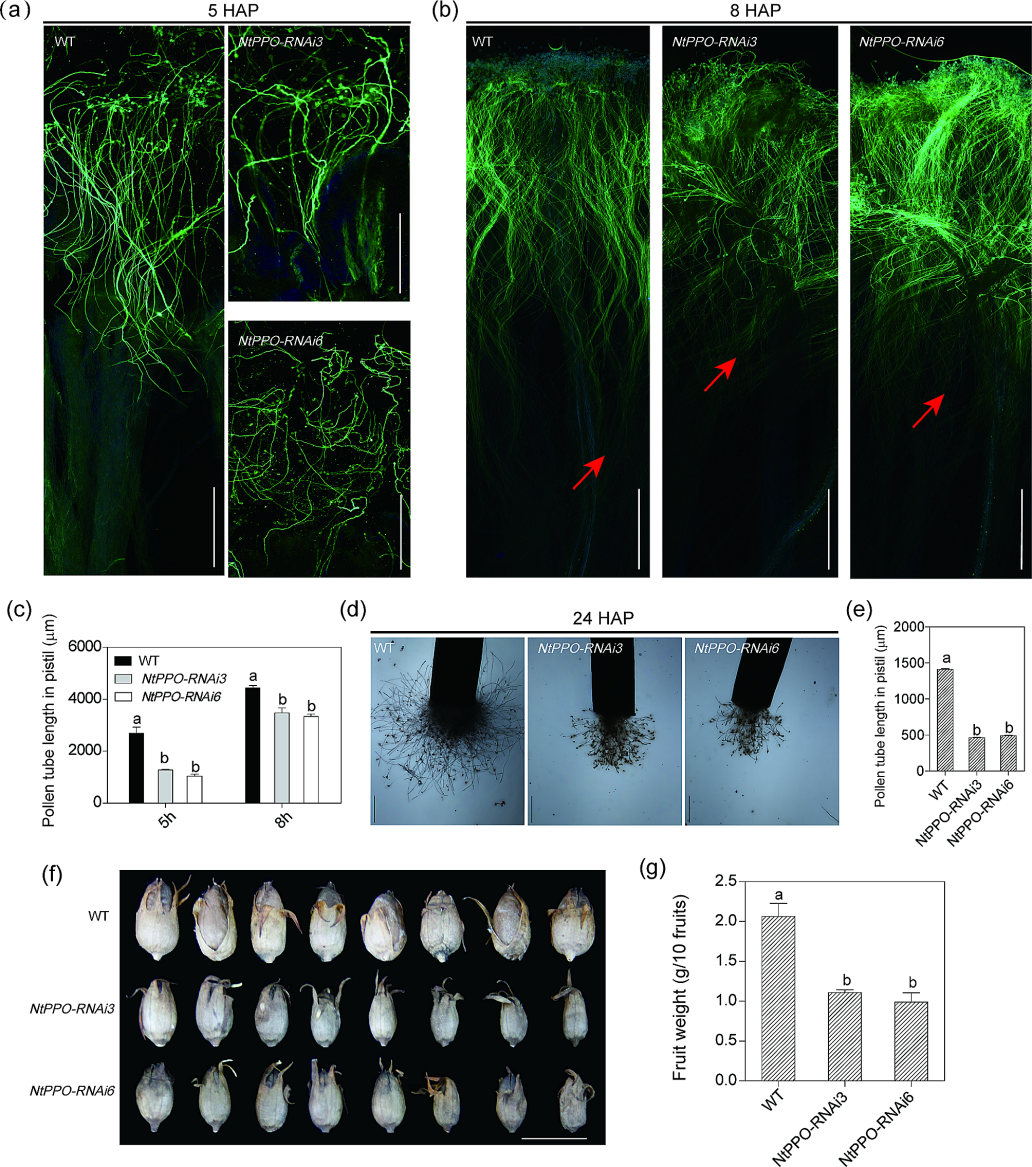
*NtPPOs* affect pollen growth in the pistil and fruit weights. (**a-b**) The pollen tube in the style at 5 and 8 HAP. HAP, hours after self-pollination. Bars = 500 μm. (**c**) Quantification of pollen tube length in the self-pollinated pistil. *n* = 15 pollinated pistils were measured across three biological replicates, and the error bar represents the standard deviation. Data were analyzed by ANOVA, *P* < 0.05. (**d-e**) The semi in vitro test of the pollen tube growth in the pistil at 24 HAP. Bars = 500 μm. *n* = 15 pollinated pistils were measured across three biological replicates, and the error bar represents the standard deviation. Data were analyzed by ANOVA, *P* < 0.05. (**f-g**) Mature fruits and quantification of fruit weight. Bars = 1 cm. *n* = 30 fruits were measured across three biological replicates; each group contains 10 fruits. Error bars indicate standard deviation of three biological replicates. Data were analyzed by ANOVA, *P* < 0.05

To investigate the function of *NtPPOs* in plant reproduction, we have constructed *RNAi* lines against all *NtPPOs* in *N. tabacum* [26]. WT and two *NtPPOs* silencing lines (*NtPPO-RNAi3/6)* were self-pollinated 5 and 8 h, and stained with aniline blue and observed on an optical microscope. The results showed that *NtPPO-RNAi3/6* lines pollen tubes grew significantly less rapidly in the pistil of *NtPPO-RNAi3/6* lines than that in the WT pistil at 5 and 8 HAP (Fig. 6a, b and c). Semi in vitro test also showed that pollen tube growth rate reduced in the pistil of *NtPPO-RNAi3/6* lines compared to WT (Fig. 6d and e). The fruit weights of *NtPPO-RNAi3/6* were significantly lighter than WT (Fig. 6f and g). These data collectively suggested that *NtPPOs* are involved in pollination and fruit development.

### Purine and flavonoids compounds are accumulated in pollinated-pistils after knockdown of *NtPPOs*

**Fig. 7:**
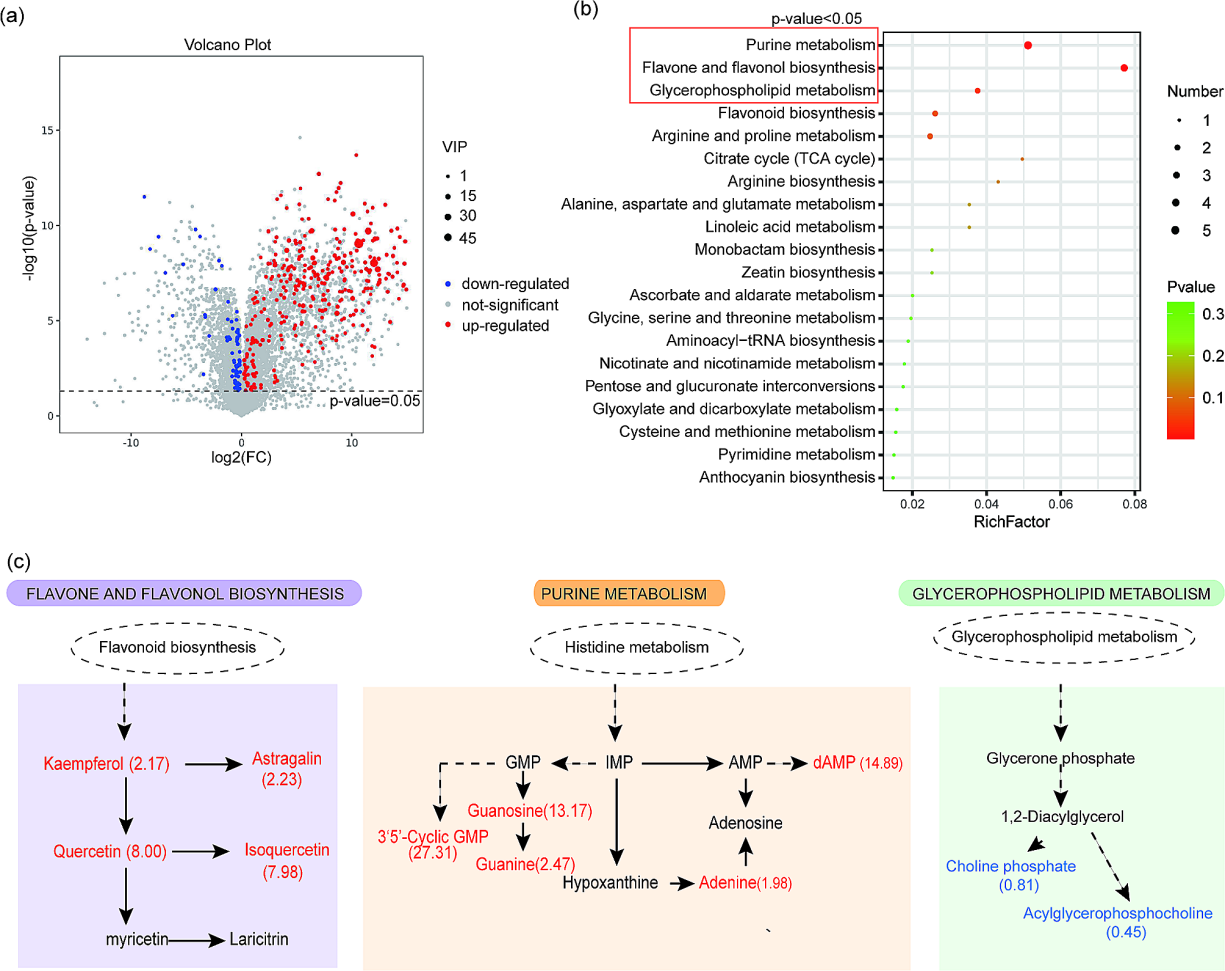
Differential metabolites analysis in pollinated-pistils after knockdown of *NtPPOs*. (**a**) Volcano plots present differential metabolites in pollinated-pistils between *NtPPO-RNAi* lines and WT. (**b**) Bubble plots show the KEGG pathway enrichment of differential metabolites in pollinated-pistils between *NtPPO-RNAi* lines and WT. Red box indicates the difference of KEGG pathway between *NtPPO-RNAi* lines and WT is significant, *P* < 0.05. (**c**) Differential metabolites in the differential significant pathway. Metabolites are colored to represent changes in abundance in the *NtPPO-RNAi* lines relative to the WT: red indicates increased abundance and up-regulation; blue indicates decrease and down-regulation; black indicates no change. Quantitative values after metabolites represent fold change (*NtPPO-RNAi* lines/wild type). Dashed arrows represent multiple enzyme-catalyzed reactions. dAMP, Deoxyadenosine monophosphate; AMP, Adenosine 5’-monophosphate; IMP, Inosine monophosphate; GMP, Guanosine monophosphate

As a class of enzymes, PPOs play a role by catalyzing substance synthesis and metabolism. Therefore, to explore the mechanism of *NtPPOs* in pollination, we used non-targeted metabolomics technology to detect the differences of metabolites in *N. tabacum* pollinated-pistils between *NtPPO-RNAi* lines and WT. The results showed that a total of 211 differential metabolites were identified, and 169 and 42 of them were accumulated and reduced respectively (Table S5, Fig. 7a). There were significant differences between *NtPPO-RNAi lines* and WT in three pathways, including purine metabolism, flavone and flavonols biosynthesis, and glycerophospholipid metabolism (Fig. 7b). Differential metabolites including dAMP, guanosine, guanine, adenine, 3 ‘5’ -cyclic GMP and kaempferol, astragalin, auercetin, isoquercetin were mainly enriched in purine metabolism pathway, and flavone and flavonols biosynthesis pathway. In phenylpropanoid biosynthesis pathway, the differential metabolites choline phosphate and acylglycerol choline phosphate were reduced (Fig. 7c). Altogether, these results suggest that NtPPOs play a role in pollination mainly through purines and flavonoid compounds.

## Discussion

Pollen development, pollination and fertilization are key processes in plant reproduction. In our previous work [26], we screened 13 *PPO* members in *Nicotiana* genome and investigated their physiological functions and regulatory mechanisms in pollen development, and found that *NtPPOs* affected pollen development by modulating flavonoids homeostasis. Moreover, we explored the expression profile and distribution of *NtPPOs* in developing anthers at transcript and protein levels, and analyzed relationship between NtPPOs and PPOs in their ancestor (*N. sylvestris* and *N. tomentosoformis*). However, the expression characteristics of *NtPPOs* in different tissues, especially in female gametophyte are not clarified, and their effects in pollination and fertilization are still unclear. In this work, we detect the expression profiles and distributions of *NtPPOs* in root, stem, leaf and female gametophyte, and explore the functions of *NtPPOs* in pollination and fertilization, and find that *NtPPOs* play an important role in pollination and fertilization by affecting the metabolism of flavonoid compounds (an important class of PPOs substrates) and purine compounds (non-PPOs substrates). In addition, we further analyse the phylogenetic relationships and compare the structural characteristics between NtPPOs and PPOs from other species.

Phylogenetic analysis and multiple sequence alignment show that PPOs in *S. lycopersicum*, *L.esculentum* and *S. tuberosum* are closely related to NtPPOs, which may be related to their membership belonging to the *Solanaceae* family. NtPPOs contain two conserved copper-binding domains with histidine residues, a His-rich region is found at the C terminus. Previous studies also reveal that PPO is a type-3 copper enzyme containing two copper ions, each coordinated by three histidine residues to form two conserved domains, which have a high degree of homology among divergent species [1, 43]. In addition to the two known copper-binding domains among various species, PPO may contain a third His-rich region at the C terminus but it is unclear if this region has any catalytic significance in the function [5, 44].

Excepting NtPPO4, the thylakoid transfer domain is detected in 12 out of 13 NtPPOs. NtPPOs are predicted to mainly localize in the chloroplast, and this result is consistent with previous studies [45, 46]. Meanwhile, NtPPOs may be diversely localized in other cell compartments or organelles. For example, IF analysis show that NtPPOs signals are detected in the tip of growing pollen tubes (cells without chloroplast), and at the periphery of the embryo sac (extracellular spaces). However, no chloroplasts are distributed in pollen cell and embryo sac. Previous studies also reveal that the diversity of PPOs location, such as AmAS1 (a PPO in *Antirrhinum majus* ) and PtrPPO13 (a PPO in poplar) have been found to locate in the lumen of vacuole [47], AcPPO in *Annona cherimola* is located in Golgi apparatus [48, 49]. Furthermore, several plant PPOs are not located in the chloroplast, and the subcellular localization of these PPO remains undetermined [50].

Differential expression patterns of *PPOs* in different tissues have been observed in the case of eggplant [51], tomato [52], and potato [53], indicating that different *PPO* members may be involved in different biological functions depending on the tissue. In this study, RT-qPCR analysis reveals that *NtPPO9* and *NtPPO10* are accumulated in the pistil and mature anther, particularly *NtPPO10*. Previous study found a *PPO* member, *tobP1*, had been isolated from a tobacco stigma/style cDNA library, which has been reported to be present exclusively in flower organs (petals, stamens, and, predominantly, pistils) [25]. Amino acid sequence alignment shows that tobP1 corresponds to NtPPO10. Moreover, NtPPO signals are detected in the pollen tube, TTS and the embryo sac, revealing their potential function in regulating pollen tube growth in the pistil.

The interaction between the pollen and the style is similar to that of bacterial defense responses—namely, pollen grains might induce comprehensive pathogen defense-like responses during self-/cross-pollination [24]. In this study, higher *NtPPOs* expression levels in the pollinated-pistil are also detected, indicating that pathogen defense-like responses are activated, which is consistent with the report that the expression of defensin and resistance protein genes is high at the beginning of compatible pollination [54]. However, the activity of NtPPOs is increased at 15 HAP, unsynchronized with *NtPPOs* expression, while in the developing anther, the activity and expression of *NtPPOs* are at the same pace [26]. The lag time between the activity and expression of *NtPPO*s may be due to a pronounced persistence of PPOs. This same phenomenon has been observed in apricot where PPO protein is still present and active at a late stage of fruit development, but its mRNA is no longer detectable [55]. In contrast, in tomato, the PPO accumulation patterns mirrors the *PPO* transcript patterns [56].

PPO is widely involved in accumulating and cycling secondary metabolites, such as phenolic monomers (mainly phenols and flavonoids), quinines, aurones, and tyramines, which may directly function in pollen tube growth [14, 21, 57]. In this work, *NtPPOs* knockdown affected pollen growth in post-pollinated pistils and fruit weight, and purines and flavonoids accumulated in pollinated-pistils of the *NtPPOs-RNAi* lines, indicating that NtPPOs play an important role in pollination and fertilization, and may directly or indirectly affect the metabolism of flavonoid compounds and purine compounds. Some other reports also found that PPO is involved in biological processes by the metabolism of its substrates, such as flavonoids play an important role in regulating pollen germination and growth by regulating ROS levels [58], caffeic acid (one of PPO substrates) causes *P. patens* growth reduction [59]. Moreover, it was reported that the substances induced by immune reaction have a promoting effect on pollen hydration, germination, growth, and orientation [60], although some substances have inhibitory effects on pollen tubes in a time dependent way [61]. Normally, the phenolic monomers are spatially separated from the PPOs at various subcellular sites [62]. The mechanism of *NtPPOs* and their substrates in pollinated–pistils is worth to be investigated in the future.

## Conclusion

Taken together, we analyzed the phylogenetic relationship, conserved domain and cis-acting elements of *NtPPOs*, and explored their expression, distribution, and localization in the reproductive tissues of *Nicotiana*. After knockdown of *NtPPOs*, the pollen tube growth rate in the pistil and fruit weights are reduced, and the purine and flavonoids compounds are accumulated in pollinated-pistils. Our experimental results have demonstrated that *NtPPOs* are important regulators to participate in pollination, and would offer new insights into the role of *PPOs* in reproduction.

### Electronic supplementary material

Below is the link to the electronic supplementary material.

Supplementary Material 1

Supplementary Material 2

Supplementary Material 3

Supplementary Material 4

Supplementary Material 5

## Data Availability

The data that support the findings of this study are available in the supporting information of this article.
