# Supplementary material for: The PPO family in Nicotiana tabacum is an important regulator to participate in pollination
Source: BMC Plant Biol. 2024 Feb 9;24:102. doi: 10.1186/s12870-024-04769-3 (PMC10854075; doi:10.1186/s12870-024-04769-3)
Supplement: Supplementary file 1 — Supplementary Material 1 [file 12870_2024_4769_MOESM1_ESM.docx]

**Table S1 The primers used in this study**

| **Purpose of use** | **Primer name** | **Sequence(5'-3')** | **Amplification**  **product length**  **(bp)** |
| --- | --- | --- | --- |
| Gene expression | NtPPO1 | F-GAGGTCTTTATGGTGCTGCTAC | 117 |
|  |  | R-GTGCTGCACCTGGTGGTAGA |  |
|  | NtPPO2 | F-AGATTTGCCACCAGGAGCAG | 101 |
|  |  | R-GAGTACGGAGGGTGGTTGAC |  |
|  | NtPPO3 | F-GTGCTGCTACTCTTGTTGGTG | 103 |
|  |  | R-GCTGCACCTGGTGGCAAA |  |
|  | NtPPO4 | F-ACCAATGCCATCTCTTCATCAG | 141 |
|  |  | R-CTTGCATGAAACTTGGAAACGA |  |
|  | NtPPO5 | F-ACGAACCAACATCCACACCA | 105 |
|  |  | R-GTGGGAATACCTTGCTGGC |  |
|  | NtPPO6 | F-AGGTGAATCCAAGTTCAGTTAAGC | 102 |
|  |  | R-TCCTTGATGTAGCTGGCCTC |  |
|  | NtPPO7 | F-TGCGGTAACTGTGGTTCCAA | 128 |
|  |  | R-AGGAAGGACCCCTAAGACACA |  |
|  | NtPPO8 | F-ACATGTTTGATGTCGAAGGTACAA | 120 |
|  |  | R-GGTTTGCCAAAGAAGAGCGA |  |
|  | NtPPO9 | F-TGCGGTAACTGTGGTTCCAA | 137 |
|  |  | R-ACAAAAGTGCACACACTGCG |  |
|  | NtPPO10 | F-GACACCATTGCGGTAACTG | 135 |
|  |  | R-GCACACACTGCGTTCAGATAA |  |
|  | NtPPO11 | F-TGATGGCGTGCCTAAAATACC | 188 |
|  |  | R-CTGTCGGCAACGCCTTCATA |  |
|  | NtPPO12 | F-ATGCACGTTCGGTTCAGTCA | 116 |
|  |  | R-TGCGTCTTTCTCCTGCTGAG |  |
|  | NtPPO13 | F-ACGTTCAGTTCCTCCAGTTACC | 163 |
|  |  | R-GTGCTTTCGCTGATCGATG |  |
|  | Ntubc2 | F-CTGGACAGCAGACTGACATC | 56 |
|  |  | R-CAGGATAATTTGCTGTAACAGATTA |  |
