# Supplementary material for: The PPO family in Nicotiana tabacum is an important regulator to participate in pollination
Source: BMC Plant Biol. 2024 Feb 9;24:102. doi: 10.1186/s12870-024-04769-3 (PMC10854075; doi:10.1186/s12870-024-04769-3)
Supplement: Supplementary file 2 — Supplementary Material 2 [file 12870_2024_4769_MOESM2_ESM.docx]

| **Gene name** | **GenBank**  **accession number** | **Origin** | **Reference** |
| --- | --- | --- | --- |
| *pMD-PPO2* | AAK56323.1 | Fugi apple | (Kim et al., 2001) |
| *pAPO5* | AAA69902.1 | apple | (Boss et al., 1995) |
| *PPO A/A’* | Q08303.2 | *Solanum lycopersicum* | (Newman et al., 1993) |
| *PPOB* | NP_001296326.1 | *Solanum lycopersicum* |  |
| *PPO C* | Q08305.1 | *Solanum lycopersicum* |  |
| *PPO D* | NP_001334885.1 | *Solanum lycopersicum* |  |
| *PPO E* | Q08307.1 | *Solanum lycopersicum* |  |
| *PPO F* | Q08296.1 | *Solanum lycopersicum* |  |
| *LePPO* | AAB22610.1 | *Lycopersicon esculentum* | (Shahar et al., 1992) |
| *StPPO* | AAA02879.1 | *Solanum tuberosum* | (Hunt et al., 1993) |
| *VfPPO* | CAA77764.1 | *Vicia faba* | (Cary et al., 1992) |
| *VvPPO* | CAA81798.1 | *Vitis vinifera* | (Dry and Robinson, 1994) |
| *PaPPO* | AAC28935 | *Prunus armeniaca* | (Chevalier et al., 1999) |
| *PtPPO* | AEH41425.1 | *Populus trichocarpa* | (Lan and C Peter, 2011) |

**Table S2 PPOs in different species.**
