# Supplementary material for: The PPO family in Nicotiana tabacum is an important regulator to participate in pollination
Source: BMC Plant Biol. 2024 Feb 9;24:102. doi: 10.1186/s12870-024-04769-3 (PMC10854075; doi:10.1186/s12870-024-04769-3)
Supplement: Supplementary file 3 — Supplementary Material 3 [file 12870_2024_4769_MOESM3_ESM.docx]

**Table S3 Function prediction of the cis-elements detected in the *NtPPOs* promoters.**

| **Cis-acting elements** | | **Function prediction** |
| --- | --- | --- |
| Plant hormones-responsive elements | ABRE | abscisic acid responsiveness |
|  | AuxRR-core | auxin responsiveness |
|  | TGA-element | auxin responsiveness |
|  | GARE-motif | gibberellin responsiveness |
|  | CGTCA-motif | MeJA responsiveness |
|  | TCA-element | salicylic acid responsiveness |
| Stress-inducible and defense-related elements | TGACG-motif | MeJA-responsiveness |
|  | P-box | gibberellin responsiveness |
|  | as-1 | auxin,salicylic acid and light-responsive element |
|  | ERE | Ethylene responsiveness |
|  | TC-rich repeats | efense and stress responsiveness |
|  | MBS | drought inducibility |
|  | Myb | stress responsiveness |
|  | MYC | stress responsiveness |
|  | WUN-motif | wound responsiveness |
|  | W box | Salicylic acid, gibberellin, drought and sugar response elements, WRKY binding elements |
|  | WRE3 | wound responsiveness |
|  | STRE | pressure responsiveness |
|  | DRE1 | Synergistic effect with ABRE to respond to drought, high salt, low-temperature and ABA |
|  | ARE | anaerobic induction |
|  | GC-motif | anoxic specific inducibility |
|  | LTR | low-temperature responsiveness |
